# Respiratory Syncytial Virus, Influenza and SARS-CoV-2 in Homeless People from Urban Shelters: A Systematic Review and Meta-Analysis (2023)

**DOI:** 10.3390/epidemiologia5010004

**Published:** 2024-01-31

**Authors:** Matteo Riccò, Antonio Baldassarre, Silvia Corrado, Marco Bottazzoli, Federico Marchesi

**Affiliations:** 1 AUSL–IRCCS di Reggio Emilia, Servizio di Prevenzione e Sicurezza Negli Ambienti di Lavoro (SPSAL), Local Health Unit of Reggio Emilia, 42122 Reggio Emilia, Italy; 2 Department of Experimental and Clinical Medicine, University of Florence, 50134 Florence, Italy; 3 ASST Rhodense, Dipartimento della Donna e Area Materno-Infantile, UOC Pediatria, 20024 Milan, Italy; scorrado@asst-rhodense.it; 4 Department of Otorhinolaryngology, APSS Trento, 38122 Trento, Italy; marco.bottazzoli@apss.tn.it; 5 Department of Medicine and Surgery, University of Parma, 43126 Parma, Italy; federico.marchesi@unipr.it

**Keywords:** RSV, viral pneumonia, differential diagnosis, homelessness, influenza, SARS-CoV-2

## Abstract

Homeless people (HP) are disproportionally affected by respiratory disorders, including pneumococcal and mycobacterial infections. On the contrary, more limited evidence has been previously gathered on influenza and severe acute respiratory syndrome coronavirus 2 (SARS-CoV-2), and very little is known about the occurrence of human respiratory syncytial virus (RSV), a common cause of respiratory tract infections among children and the elderly. The present systematic review was designed to collect available evidence about RSV, influenza and SARS-CoV-2 infections in HP, focusing on those from urban homeless shelters. Three medical databases (PubMed, Embase and Scopus) and the preprint repository medRxiv.org were therefore searched for eligible observational studies published up to 30 December 2023, and the collected cases were pooled in a random-effects model. Heterogeneity was assessed using the I^2^ statistics. Reporting bias was assessed by funnel plots and a regression analysis. Overall, 31 studies were retrieved, and of them, 17 reported on the point prevalence of respiratory pathogens, with pooled estimates of 4.91 cases per 1000 HP (95%CI: 2.46 to 9.80) for RSV, 3.47 per 1000 HP for influenza and 40.21 cases per 1000 HP (95%CI: 14.66 to 105.55) for SARS-CoV-2. Incidence estimates were calculated from 12 studies, and SARS-CoV-2 was characterized by the highest occurrence (9.58 diagnoses per 1000 persons-months, 95%CI: 3.00 to 16.16), followed by influenza (6.07, 95%CI: 0.00 to 15.06) and RSV (1.71, 95%CI: 0.00 to 4.13). Only four studies reported on the outcome of viral infections in HP: the assessed pathogens were associated with a high likelihood of hospitalization, while high rates of recurrence and eventual deaths were reported in cases of RSV infections. In summary, RSV, influenza and SARS-CoV-2 infections were documented in HP from urban shelters, and their potential outcomes stress the importance of specifically tailored preventive strategies.

## 1. Introduction

Because of the challenges represented by poor environmental conditions with cold and heat stress [1], a high proportion of smoking habits, addiction to alcohol and/or illicit drugs and mental health issues [2,3,4,5,6], people without adequate housing or without permanent residence (i.e., ill-housed or homeless people, HP) are collectively considered a medically vulnerable population [1]. According to available estimates [7,8], the longer a person is homeless, the more likely it is that this condition will result in increased morbidity and mortality [9], with reduced life expectancy and mortality rates that exceed those of the general population by 3 to 13 times [7,8,10,11,12,13]. Following the COVID-19 pandemic, the ongoing migratory crisis and local conflicts (e.g., the Syrian civil war, the war in Ukraine, etc.), this social problem is increasingly affecting all high-income countries [14,15,16], representing a global health problem [1,15,16]. For instance, in the European Union/European Economic Area (EU/EEA) alone, the number of HP has nearly doubled in the last 10 years, up to around 1,000,000 people [14,15], while in the USA, the overall estimates point to more than 580,000 people by 2023, reaching record highs in the history of data collection [16].

Homeless shelters are temporary residences for HP, providing safety conditions and protection from exposure to the weather [1,4,15,16]. Homeless shelters are often crowded, and the shared living spaces and rooms guarantee only limited access to hygiene facilities and supplies, eventually impairing a HP’s ability to cope with personal hygiene requirements [4,12,15,17,18]. Despite the efforts made by managing authorities, homeless shelters are therefore characterized by a high circulation and transmission of respiratory pathogens [2,3,4,5,6,19,20,21], including *Mycobacterium tuberculosis* [22,23,24,25], *Streptococcus pneumoniae* [2,26,27,28,29], *Neisseria meningitidis* [30,31,32,33] and *Corynebacterium diphtheriae* [34]. In other words, HP and homeless shelters represent likely targets for outbreaks of highly diffusive respiratory viruses such as influenza, SARS-CoV-2 and respiratory syncytial virus (RSV), all of which are characterized by similar transmission characteristics, clinical manifestations and cumulative disease burden [35,36]. For instance, according to estimates from the United States Centers for Disease Prevention and Control (CDC), during the winter season in 2022, influenza caused at least 27 million illnesses, around 300,000 hospitalizations and 19,000 deaths in the United States alone [36]. Nonetheless, SARS-CoV-2 still causes high rates of medical consultation for respiratory illness and high rates of test positivity in cases of severe acute respiratory infections [37]. Even though on 5 May 2023, the World Health Organization (WHO) Emergency Committee on COVID-19 recommended to the Director-General, who accepted the recommendation, that SARS-CoV-2 no longer fit the definition of a Public Health Emergency of International Concern [38], SARS-CoV-2 still remains of global concern. For example, more than 3000 new deaths were globally reported between 20 November and 17 December 2023 [39]. While data on the occurrence and carriage rate among HP of influenza and SARS-CoV-2 have been extensively collected [5,19,20,21,40,41,42,43,44,45], particularly during the early stages of the COVID-19 pandemic, relatively little evidence is available on RSV.

RSV is an enveloped and pleomorphic, negative-sense, single-stranded RNA virus of medium size (120 to 300 nm diameter) that belongs to the genus orthopneumovirus (family *Pneumoviridae*) [46,47,48,49]. As a highly contagious pathogen, before the SARS-CoV-2 pandemic, RSV was acknowledged as being the single most common viral cause of lower respiratory tract infections (LRTIs) [48]. It has been estimated that before 2020, up to 33 million cases occurred each year in the world [47,50]; during the COVID-19 pandemic, physical distancing and non-pharmaceutical interventions led to a stark decrease in the global rates of RSV-associated hospitalization (−79.7% in high-income countries, −13.8% in upper-middle-income countries) [51,52,53,54,55,56]. Nonetheless, these figures are affected by a certain degree of underestimation. On the one hand, RSV usually causes self-limited upper respiratory tract infections [46,47,57], which in most cases only lead to the development of mild respiratory symptoms [46,47,48], with a reduced proportion of incident cases evolving to LRTIs [58]. Secondly, up to 90% of incident cases are not properly reported to competent health authorities as diagnostic testing for RSV is not regularly performed [59,60,61].

As nearly all children are usually infected by RSV before their second year of age [46,62,63], it has been mostly regarded as a pediatric pathogen [64,65,66,67]. However, a growing body of evidence suggests that RSV infections are not limited to pediatric-age subjects [61,68], causing a substantial burden of disease in all fragile subjects [69,70], irrespective of their actual age. For example, a recent report from the EU/EEA suggests that on average, more than 150,000 RSV-associated hospitalizations occur annually among adults in the EU alone, and 92% of these hospitalizations occur in adults aged 65 years or older [53]. Adults and elderly people may also develop a high rate of complications due to RSV-related LRTIs. In a study from the United States reporting on the timeframe February 2022–March 2023, the hospitalizations for RSV in adults aged ≥ 60 years (N. = 304) were several times less frequent than those associated with SARS-CoV-2 (N. = 4734) or seasonal influenza virus (N. = 746) infections, but they were associated with a more severe outcome, with a higher occurrence of ICU admission and death, even compared to SARS-CoV-2 and seasonal influenza [71]. More precisely, the odds for hospitalization and death due to RSV compared to SARS-CoV-2 were estimated as an adjusted odds ratio (aOR) of 1.49 (95% confidence interval (95%CI): 1.13 to 1.97) and an aOR of 1.39 (95%CI: 0.98 to 1.96), respectively. Similarly, the odds for RSV-related hospitalizations and deaths compared to seasonal influenza were estimated as an aOR of 1.55 (95%CI: 1.11 to 2.19) and 2.08 (95%CI: 1.33 to 3.25), respectively.

Even though interventions with influenza and SARS-CoV-2 vaccination campaigns have already been put in place for preventing severe outcomes among fragile populations [72,73,74], the recent licensing of new and effective vaccines and monoclonal antibodies against RSV [61,75,76,77,78,79,80,81] suggests that the collection and accurate analysis of data on the epidemiology of and clinical presentation of influenza, SARS-CoV-2 and RSV among HP could be important for decisions around potential vaccine delivery and mitigation strategies in shelter settings. Specifically, we focused the present systematic review and meta-analysis on the following research questions: (1) What is the reported occurrence of RSV, influenza and SARS-CoV-2 in HP from homeless shelters? (2) Is the reported occurrence of RSV associated with an increased case fatality ratio compared to other viral respiratory pathogens?

## 2. Materials and Methods

### 2.1. Research Concept

We designed a systematic review and meta-analysis in accordance with the “Preferred Reporting Items for Systematic Reviews and Meta-Analysis” (PRISMA) statement [82] (see Table S1). As a preliminary step, it was registered into the PROSPERO database, an international repository of prospectively registered systematic reviews in health and social care, welfare, public health, education, crime, justice and international development (progressive registration number: CRD42023475548).

The research concepts were defined by means of the “PECO” strategy (i.e., patient/population/problem; exposure; control/comparator; outcome) [83,84] (Appendix A, Table A1). More precisely, we assessed among individuals being assisted in urban shelters for homeless people (P) the occurrence (i.e., prevalence and/or incidence) of RSV (E) in children and adults compared to influenza and SARS-CoV-2 infections (C). We eventually collected corresponding health outcomes, including requests for medical assistance, hospitalizations and deaths, where available (O).

### 2.2. Research Strategy

The search strategy was designed through a combination of specifically designed search strings and was performed across three databases (i.e., PubMed, by means of Medical Subject Heading (MeSH) terms; EMBASE; and Scopus) and the preprint repository medRxiv (Appendix A, Table A2).

### 2.3. Screening

For the aims of the present review, documents were considered eligible if their prospective or retrospective design included data on the prevalence, incidence and/or outcome of RSV, influenza and/or SARS-CoV-2 infections in individuals from urban homeless shelters.

Working definitions for HP and homeless shelter are provided in Appendix A, Table A3: for the aims of the present review, persons living on the streets, in open spaces or cars or in severely inadequate and insecure housing, such as residents of informal settlements, even though included in the definitions of homelessness described by the United Nations Human Rights Office, were not included [14,15].

Only studies based on Real-Time Quantitative Polymerase Chain Reaction (RT-qPCR) were included in the qualitative and quantitative summary.

The following exclusion criteria were then applied:

(1) The full text was not available either through online repositories or through inter-library loan or its main text was written in a language other than English, Italian, German, French, Spanish, Portuguese or Farsi;
(2) The study was designed as a case report, a case series, or a review/systematic review;
(3) The study did not mention the geographical setting or corresponding timeframe;
(4) There was a lack of detailed reporting of the sampling approach, including the respective inclusion/exclusion criteria for the collection of samples from potentially participating HP;
(5) Studies carried out in refugee camps and shelters: we deliberately ruled out this specific subgroup of HP because of the presumptively high proportion of female individuals, children and adolescents compared to those usually reported by urban shelters [9];
(6) The total number of sampled HP was not provided;
(7) The laboratory diagnosis of respiratory infections was performed using methods other than RT-qPCR (e.g., clinical features, imaging, seroprevalence studies, etc.).

When a retrieved article provided data on duplicated patients and/or series, on HP from settings other than urban shelters and/or on workers from the shelters, those data were removed from the qualitative and quantitative analysis when possible. If only cumulative data were provided, the article was then removed.

Articles fulfilling the inclusion criteria but not included in the exclusion criteria were initially title-screened to ascertain their relevance to the research question. The abstracts of the items positively title-screened were then analyzed [82,85], and all the entries that were found to be consistent with the aims of the research question were eventually full-text screened and independently rated by two investigators (AB, FM). All potential disagreements were either resolved by consensus between the investigators or, where this was not reached, through the input of the chief investigator (MR).

### 2.4. Summary of Retrieved Data

The data extracted included:

(a) The settings of the study: country, region, timeframe of the study and/or observation period(s);
(b) The number of HP potentially included in the estimate(s);
(c) Where available, demographic data and characteristics of the sampled HP (i.e., age, gender, abuse of alcohol, smoking history, abuse of intravenous (IV) drugs, abuse of cannabis);
(d) The number of collected samples (total);
(e) The number of samples with a positive RT-qPCR diagnosis for RSV, influenza and SARS-CoV-2.

When a single study reported on two or more timeframes, the data were separately reported and analyzed as distinctive series.

### 2.5. Risk of Bias Analysis

Individual studies can be biased due to research practices [86,87,88], eventually impairing the validity of the quantitative evidence collected by means of the meta-analysis. To preventively assess the risk of bias (ROB) of the retrieved studies, we implemented the ROB tool provided by the National Toxicology Program’s (NTP) Office of Health Assessment and Translation (OHAT) [88,89]. The OHAT ROB was preferred over other similarly designed instruments as it neither applies an overall rating for each study nor requires that studies reasonably affected by a substantial ROB be removed from the pooled analyses, which could lead to underestimating the health effects of the considered exposure [89]. By design, the OHAT ROB focuses on the internal validity of a given study by weighting the following sources of bias: participant selection (D1), confounding factors (D2), attrition/exclusion (D3), detection (D4) and selective reporting (D5), as well as other sources of bias (D6). All sources of bias are rated from “definitely low,” “probably low,” “probably high,” to “definitely high” regarding the likelihood they do or do not compromise the association between an exposure and the reported outcome.

### 2.6. Data Analysis

The studies were initially categorized into (a) prevalence studies; (b) incidence studies; and (c) studies providing the outcome of sampled infections (i.e., outcome studies). The prevalence rates for RSV, seasonal flu and SARS-CoV-2 were initially calculated as the number of positive specimens over the whole number of collected samples. If a study did not include raw data, either as prevalent cases or a reference population, such figures were reverse calculated from available information. All estimates were initially reported as numbers per 100 specimens. Incidence rates were calculated by a cumulative calculation of the person-month observation time provided by each study. Moreover, by using the prevalence estimates for influenza as the reference groups, Risk Ratios (RRs) and their corresponding 95% confidence intervals (95%CI) were calculated in a bivariate analysis for RSV and SARS-CoV-2. The RRs for RSV, influenza and SARS-CoV-2 infections in the post-pandemic timeframe (after 1 January 2020) vs. the pre-pandemic timeframe (before 31 December 2019) were similarly calculated. The odds ratios (ORs) for the outcome variables (i.e., assessment by a healthcare provider, hospitalization, ICU admission, death) were similarly calculated in a bivariate analysis.

Pooled estimates for incidence and prevalence were calculated through a random effect model (REM) meta-analysis of the retrieved studies, and the data were reported as estimates for all the retrieved studies for pre-pandemic and pandemic studies. Moreover, the pooled ORs for RSV and SARS-CoV-2 infections were similarly calculated, and influenza was considered the reference group. A REM was preferred over a fixed-effect model as it is more effective in dealing with the presumptive variation in study outcomes and ascertaining the genuine differences underlying the results of studies (heterogeneity) [90,91]. The inconsistency of effects between the included studies was defined as the percentage of total variation across the studies likely due to heterogeneity rather than chance [86] and was quantified by the calculation of the I^2^ statistic. I^2^ estimates were classified as follows: 0 to 25%, low heterogeneity; 26% to 50%, moderate heterogeneity; and ≥50%, substantial heterogeneity. The 95%CIs of the I^2^ estimates were provided to cope with the potential small size of the meta-analyses [86].

A sensitivity analysis (i.e., the study of how the uncertainty in the output of a mathematical model or system can be apportioned to different sources of uncertainty in its inputs) was performed to evaluate the effect of each study on the pooled estimates by excluding one study at a time. Any significant changes in the pooled estimates were reported. The potential publication bias was ascertained through the calculation of contour-enhanced funnel plots, and their asymmetry was eventually assessed by means of Egger’s test [82,92]. A small study bias was eventually assessed by generating corresponding radial plots.

All calculations were performed in R (version 4.3.1) [93] and Rstudio (version 2023.06.0 Build 421; Rstudio, PBC; Boston, MA, USA) software by means of the packages meta (version 6.5-0) and fmsb (version 0.7.5). A Prisma2020 flow diagram was designed by means of the PRISMA2020 package [94].

## 3. Results

### 3.1. Descriptive Analysis

**Figure 1:**
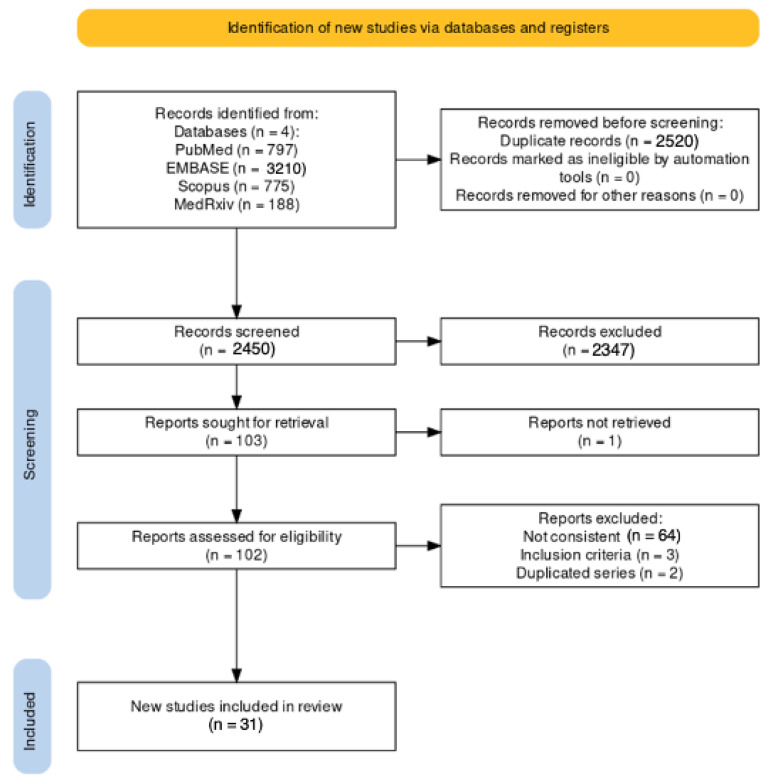
Flowchart of included studies.

A total of 4970 entries were identified in the database searches (Figure 1; Appendix A, Table A2); the majority (3210, 64.59%) were identified in EMBASE, followed by PubMed (797, 16.04%), Scopus (775, 15.59%) and medRxiv (188 entries, 3.78%).

As 2520 of the retrieved reports were duplicates (50.70%), 2450 of them were title- and abstract-screened (49.30%), with the subsequent removal of 2347 further records (47.22% of the initial sample). The remaining 103 entries were sought for retrieval (2.07%) and assessed for their eligibility. One record was not retrievable and was therefore removed from the analyses [95]. A total of 102 articles were therefore full-text-reviewed; of them, 66 were removed from the analyses as they were not consistent with the research topic, while 3 further reports were removed for not fulfilling the inclusion criteria, and 2 reports were removed for including cases otherwise described in other studies [19,20,21,96,97]. The final sample included a total of 31 studies (0.62% of the initial sample); of them, 17 were prevalence studies [4,5,6,17,18,42,98,99,100,101,102,103,104,105,106,107,108], 12 studies reported on the incidence of respiratory pathogens [19,20,21,44,109,110,111,112,113,114,115,116] and the remaining 2 studies reported on the outcome of respiratory infections in HP [117,118]. As two incidence studies also included outcome data, a total of four outcome studies were ultimately retrieved [19,112].

A detailed description of the prevalence studies is provided in Table 1, while the incidence studies are summarized in Table 2, and the outcome studies are included in Table 3.

### 3.2. Characteristics of Prevalence Studies

The retrieved studies reported data from February 2005 [17] to December 2021 [21]. As the papers by Thiberville et al., 2014 [18], Ly et al., 2019 [4], Mosites et al. [105], Storgaard et al. [104], Kiran et al. [42], Ly et al. [5] and Oette et al. [99] included multiple timeframes, and the study by Mosites et al. [105] encompassed a series of cases otherwise reported by Baggett et al. [108] (that were therefore removed from the analyses), a total of 28 series and 8430 HP were ultimately included in the analyses.

The largest number of studies were performed in France (6 out of 17 studies, 35.29%) [4,5,17,18], mostly in the area of Marseille, for a total of 1784 samples (21.39% of all the samples), while the largest share of participants were recruited in the United States (2639 HP, 31.64%), followed by Belgium (1985 HP, 23.80%) [103]. A single study from Canada reported on 1000 HP (11.99%) [112], while two studies from Germany included a total of 485 HP (5.82%) [99,100]. Furthermore, 436 HP (5.23%) were included from a single study performed in Denmark [104], and 138 HP from a single study from The Netherlands (1.65%) [101].

The demographic data were irregularly available: where provided, the mean age ranged from 40.4 years ± 15.6 [6] to 51.6 years ± 12.8 [108], while the median age ranged from 41 (range: 7 to 76) [17] to 54 (interquartile range: 37 to 64) [98]. With the notable exception of a study by Storgaard et al. [104], which mostly included HP of the female gender, the majority of the sampled HP were of the male gender (pooled sample: 76.51%; range: 67.91% to 100%). The country of origin was reported for 1522 HP, and most of them (93.69%) were foreign-born, but this information was reasonably underestimated. In fact, the reports by Ly et al., 2021 [5] did not dichotomize French-born HP from individuals born in other EU/EEA countries. Similarly, data on smoking history, alcohol abuse, abuse of cannabis and IV drugs were inconsistently provided. For instance, data on smoking history were provided for 1203 out of the 8350 sampled HP. Of them, 61.41% were active smokers. Similarly, abuse of alcohol, cannabis and IV drugs was reported in 24.61%, 23.40% and 2.08% of cases where this information was provided.

Overall, five studies included estimates about RSV infection for a total of 1628 potential samples. Of them, four also included estimates on influenza (1273 potential samples). In total, 14 out of 17 studies included estimates on SARS-CoV-2 prevalence among HP for 7375 actual samples.

**Table 1:** Summary of collected prevalence studies.

Study	Country	Timeframe	Potential Sample (N.)	Total Sample (n./N., %)	Age (Years)	Males (n., %)	FB People (n.,%)	Smoking History (n., %)	Alcohol Consumption (n., %)	Abuse of Cannabis (n.,%)	Abuse of IV Drugs (n.,%)	Sampled Respiratory Viruses
Badiaga et al., 2009 [17]	France (Marseille)	1 February 2005 3 February 2005	540	221 (40.92%)	Median: 41 Range: 7 to 76	208 (94.11%)	139 (62.90%)	169 (76.47%)	77 (34.84%)	45 (20.36%)	4 (1.81%)	RSV, Flu
Thiberville et al., 2014 [18]	France (Marseille)	1 February 2010–4 February 2010	540	108 (21.60%)	Mean: 48.8 SD: 17.4	95 (87.96%)	NA	67 (62.04%)	24 (22.22%)	16 (14.81%)	1 (0.93%)	RSV, Flu
		1 February 2011–3 February 2011	540	157 (29.07%)	Mean: 46.7 SD: 16.8	142 (90.44%)	NA	90 (57.32%)	32 (20.38%)	28 (17.83%)	5 (3.18%)	
Ly et al.,2019 [4]	France (Marseille)	17 February 2015	600	125 (20.83%)	Mean: 43.5 SD: 16.0	479 (100%)	408 (85.18%)	293 (61.17%)	52 (10.86%)	75 (15.66%)	2 (0.42%)	RSV, Flu
		7 February 2016–10 March 2016	600	156 (26.00%)								
		6 February 2017–8 February 2017	600	198 (33.00%)								
Baggett et al., 2020 [108]	USA (Boston, MS)	2 April 2020– 3 April 2020	430	408 (94.88%)	Mean: 51.6 SD: 12.8	292 (67.91%)	NA	NA	NA	NA	NA	SARS-CoV-2
Imbert et al., 2020 [107]	USA (San Francisco, CA)	8 April 2020–9 April 2020	255	150 (58.82%)	NA	NA	NA	NA	NA	NA	NA	SARS-CoV-2
Karb et al., 2020 [106]	USA (Providence, RI)	19 April 2020–24 April 2020	302	299 (99.01%)	Mean: 47.9 Range: 18 to 85	249 (83.28%)	NA	NA	NA	NA	NA	SARS-CoV-2
Mosites et al., 2020 [105]	USA (Seattle, WA)	30 March 2020–8 April 2020 27 March 2020–15 April 2020	NA	392	NA	NA	NA	NA	NA	NA	NA	SARS-CoV-2
	USA (San Francisco, CA)	4 April 2020–15 April 2020	255	143 (56.08%)	NA	NA	NA	NA	NA	NA	NA	
	USA (Atlanta, GE)	8 April 2020–9 April 2020	NA	249	NA	NA	NA	NA	NA	NA	NA	
Storgaard et al., 2020 [104]	Denmark (Aarhus)	1 April 2020–30 April 2020	295	295 (100%)	Median: 50 95%CI: 38 to 59	116 (39.32%)	NA	NA	NA	NA	NA	SARS-CoV-2
		1 June 2020–30 June 2020	141	141 (100%)	Median: 53 95%CI: 42 to 61	57 (40.43%)	NA	NA	NA	NA	NA	
Husain et al., 2021 [98]	France (Paris)	1 March 2020–31 May 2020	137	100 (72.99%)	Median: 54 IQR: 37 to 64	65 (65.00%)	NA	35 (35.00%)	28 (28.00%)	NA	6 (6.00%)	SARS-CoV-2
Kiran et al., 2021 [42]	Canada (Toronto, ON)	23 April 2020–1 June 2020	872	504 (57.80%)	Mean: 45.8 SD: 16.3	713 (81.77%)	NA	NA	NA	NA	NA	SARS-CoV-2
		9 June 2020–23 July 2020	872	496 (56.88%)								
Ly et al., 2021 [5]	France (Marseille)	31 March 2020–6 April 2020	283	126 (44.52%)	Mean: 46.2 SD: 16.0	126 (100%)	94 (74.60%)	NA	NA	NA	NA	RSV, Flu, SARS-CoV-2
		22 April 2020–23 April 2020	283	111 (39.22%)	Mean: 48.5 SD: 15.5	111 (100%)	78 (70.27%)	NA	NA	NA	NA	
		16 July 2020	283	71 (25.09%)	Mean: 46.6 SD: 16.9	71 (100%)	52 (73.23%)	NA	NA	NA	NA	
Ly et al., 2021 [6]	France (Marseille)	26 March 2020–17 April 2020	716	411 (57.40%)	Mean: 40.4 SD: 15.6	369 (89.78%)	312 (75.91%)	NA	NA	NA	NA	SARS-CoV-2
Oette et al., 2021 [100]	Germany (Köln)	1 May 2021–31 May 2021	NA	130	>40 y.o. = 87 (66.92%)	118 (90.77%)	66 (50.77%)	NA	NA	NA	NA	SARS-CoV-2
Roland et al., 2021 [103]	Belgium (Brussels)	27 April 2020–10 June 2020	1994	1985 (99.55%)	Mean: 41.9 SD: 14.3	1345 (67.76%)	NA	NA	NA	NA	NA	SARS-CoV-2
Oette et al., 2022 [99]	Germany (Düsseldorf)	7 May 2021–16 May 2021	303	129 (42.57%)	>40 y.o. = 213 (70.20%)	268 (88.45%)	150 (49.50%)	NA	NA	NA	NA	RSV, SARS-CoV-2
		25 August 2021–18 September 2021	303	143 (47.19%)								
		11 December 2021–20 December 2021	303	83 (27.39%)								
Rowan et al., 2022 [102]	USA (Denver, CO)	2 June 2020–28 July 2020	NA	871	Median: 46 IQR: 36 to 55	716 (82.3%)	NA	NA	NA	NA	NA	SARS-CoV-2
Generaal et al., 2023 [101]	The Netherlands (Amsterdam)	3 May 2021–21 May 2021	138	138 (100%)	Median: 44 Range: 37 to 51	126 (91.30%)	127 (92.03%)	NA	83 (60.14%)	81 (58.70%)	7 (5.07%)	SARS-CoV-2

Note: FB = foreign-born; IV = intravenous drugs; FLU = influenza virus; SARS-CoV-2 = severe acute respiratory syndrome coronavirus 2; IQR = interquartile range; SD = standard deviation; 95%CI = 95% confidence interval; NA = not available.

**Table 2:** Summary of collected incidence studies.

Study	Country	Timeframe	Potential Sample (N.)	Total Sample (N./, %)	Age (Years)	Males (N., %)	FB People (N.,%)	Smoking History (N., %)	Alcohol Consumption (N., %)	Abuse of Cannabis (N.,%)	Abuse of IV Drugs (N.,%)	Total Tests (N.)	Sampled Respiratory Viruses
Ralli et al., 2021 [109]	Italy (Rome)	1 October 2020–5 June 2021	1665	1052 (63.18%)	NA	509 (48.38%)	NA	NA	NA	NA	NA	1052	SARS-CoV-2
Lindner et al., 2021 [111]	Germany (Berlin)	9 July 2020–29 July 2020	124	93 (75.0%)	Median: 47 IQR: 34 to 54	74 (79.57%)	NA	NA	NA	NA	NA	118	SARS-CoV-2
Richard et al., 2021 [112]	Canada (Toronto, ON)	1 June 2021–30 April 2022	2643	415 (15.70%)	Mean: 46.6 SD: 14.5	272 (65.54%)	169 (40.72%)	287 (69.19%)	264 (63.61%)	NA	158 (21.91%)	721	SARS-CoV-2
Berner et al., 2022 [113]	USA (Nationwide)	1 March 2020–30 November 2020	NA	11,563	NA	NA	NA	NA	NA	NA	NA	11,563	SARS-CoV-2
Chow et al. 2022 [21]	USA (Seattle, WA)	1 October 2019–31 May 2021	NA	3281	Median: 37 Range: 0.3 to 85	1979 (60.31%)	NA	1493 (45.50%)	NA	NA	NA	14,464	RSV, Flu, SARS-CoV-2
Keller et al., 2022 [115]	USA (Louisville, KY)	1 March 2019–31 December 2019 1 March 2020–31 December 2020	3911	711 (18.18%)	Mean: 43.6 SD: 16.4	NA	NA	NA	NA	NA	NA	711	SARS-CoV-2
Luong et al., 2022 [116]	Canada (Toronto, ON)	17 April 2020–31 July 2020	NA	4657	NA	NA	NA	NA	NA	NA	NA	4657	SARS-CoV-2
McCulloch et al., 2023 [20]	USA (King’s County, WA)	1 January 2019–31 May 2019	NA	649	Median: 41 Range: 0 to 97	NA	NA	NA	NA	NA	NA	825	RSV, Flu
		1 October 2019–31 May 2021	NA	3281		NA	NA	NA	NA	NA	NA	15,289	
Morrone et al., 2023 [110]	Italy (Rome)	1 June 2020–1 January 2022	NA	3061	Median: 44.6 Range: 5 to 86	1714 (55.99%)	2362 (77.16%)	NA	NA	NA	NA	5442	SARS-CoV-2
Rogers et al., 2023 [114]	USA (King’s County, WA)	1 January 2020–31 May 2021	NA	2360	Median: 37 IQR: 32.0	1484 (62.88%)	NA	1101 (46.65%)	NA	NA	NA	9846	SARS-CoV-2
Rogers et al., 2023 [44]	USA (King’s County, WA)	15 November 2019–30 April 2020 2 November 2020–30 April 2021	NA	1283	Median: 45 IQR: 24	878 (68.43%)	NA	814 (63.45%)	NA	NA	NA	1283	Flu
Rogers et al., 2023 [19]	USA (King’s County, WA)	21 January 2019–16 May 2019	NA	649	Mean: 53.1 SD: 11.3	496 (76.43%)	NA	508 (78.27%)	NA	NA	NA	825	RSV, Flu

Note: FB = foreign-born; IV = intravenous drugs; FLU = influenza virus; SARS-CoV-2 = severe acute respiratory syndrome coronavirus 2; IQR = interquartile range; SD = standard deviation; 95%CI = 95% confidence interval; NA = not available.

**Table 3:** Summary of collected outcome studies.

Study	Country	Timeframe	Sampled Population		Outcome	RSV		Influenza		SARS-CoV-2	
			HP (N.)	Non-HP (N.)		HP (n./N, %)	Non-HP (n./N, %)	HP (n./N, %)	Non-HP (n./N, %)	HP (n./N, %)	Non-HP (n./N, %)
Boonyaratanakornkit et al., 2019 [117]	USA (Seattle, WA)	July 2012– June 2017	24,452	350,220	Hospital admissions	50 (0.20%)	107 (0.03%)	137 (0.56%)	571 (0.16%)	NA	NA
Richard et al., 2021 [112]	Canada (Toronto, ON)	23 January 2020 to 31 July 2020	8451	1,266,716	Total cases	NA	NA	NA	NA	274 (3.24%)	28,430 (2.24%)
					Hospital admissions	NA	NA	NA	NA	104 (1.23%)	3685 (0.29%)
					ICU	NA	NA	NA	NA	15 (0.01%)	1053 (0.08%)
					Deaths	NA	NA	NA	NA	10 (0.01%)	730 (0.06%)
Loubiere et al., 2023 [118]	France (Marseille)	5 June 2020 to 31 March 2021	1332	NA	Total cases	NA	NA	NA	NA	192 (14.41%)	NA
					Hospital admissions	NA	NA	NA	NA	73 (5.48%)	NA
Rogers et al., 2023 [19]	USA (Seattle, WA)	21 January 2019 to 16 May 2019	649	NA	Total cases	14 (2.16%)	NA	11 (1.69%)	NA	NA	NA
					Sought healthcare	3 (0.46%)	NA	2 (0.31%)	NA	NA	NA

Note: HP = homeless people; RSV = respiratory syncytial virus; SARS-CoV-2 = sudden acute respiratory syndrome coronavirus 2; NA = not available.

### 3.3. Characteristics of Incidence Studies

The twelve included studies reported on data collected from January 2019 to December 2021, mostly from the USA (seven studies) [19,20,21,44,113,114,115], followed by Canada [112,116], Italy [109,110] (two studies each) and Germany (one study) [111], for a total of 36,531 HP. However, as five papers reported on HP from Seattle and King’s County [19,20,21,44,113,114,115], while the Italian and Canadian studies reported on cases drawn from the same area, the overall number of unique individuals included in the pooled sample cannot be determined.

Where available, the demographic data pointed to a sampled population with a mean age range from 43.6 ± 12.8 [115] to 53.1 ± 11.3 years [19]. The median age ranged between 37 years [21] and 47 years [111]. The majority of the sampled HP were of the male gender (9629 out of 14,713 HP; 65.44%), with a high proportion of individuals reporting a current smoking history (5969 out of 10,600; 56.22%). Only two studies reported on the country of origin of the sampled HP [110,112], for a total of 3476 individuals, and 2649 of them (76.21%) were foreign-born. Estimates about alcohol and IV drug abuse were only reported by Rogers et al. [112], with a prevalence of 63.61% and 21.91%, respectively.

Focusing on the reported samples, after the removal of duplicated data, a total of 64,415 encounters were included, most of them from the USA (53,982; 83.80%), followed by Italy (6494; 10.08%), Canada (5378; 8.34%) and Germany (118; 0.18%). The observation time ranged between 20 and 1340 days.

Regarding the sampled pathogens, three studies reported estimates of RSV infections, four on influenza and nine on SARS-CoV-2.

### 3.4. Characteristics of Outcome Studies

A total of four outcome studies were retrieved. Two of them included data collected from the Seattle area [19,117], while the remaining papers detailed cases from Toronto [112] and Marseille [118] (one study each). Two reports included data on both HP and the general population, for a total of 32,091 HP and 1,576,936 non-HP [117,118]. Regarding the two remaining studies, Loubiere et al. [118] reported on a total of 1332 HP tested for SARS-CoV-2 infection, while the study from Rogers et al. [19] included data on about 649 HP drawn from the larger population of HP in the King’s County area, otherwise included in other incidence studies. Three studies included data about the hospitalizations of the sampled individuals, while the study by Rogers et al. [19] only included data about people who sought healthcare advice following respiratory infections. Interestingly, the study by Boonyaratanakornkit et al. [117] only provided the proportion of hospitalized HP with a diagnosis of RSV or influenza, while the total number of HP diagnosed with those pathogens was not reported. On the contrary, the study by Richard et al. [112] reported on ICU admissions and eventual deaths following a SARS-CoV-2 infection.

Focusing on the reported pathogens, the studies by Boonyaratanakornkit et al. [117] and Rogers et al. [19] included data about RSV and influenza infections, while the studies by Loubiere et al. [118] and Richard et al. [112] only included diagnoses of SARS-CoV-2 infections.

### 3.5. Prevalence Estimates

As shown in Table 4, RSV was identified in 8 out of the 1628 sampled HP (0.49%), with a prevalence ranging from 0 in the study by Ly et al. [5] and in one of the series of studies by Thiberville et al. [18] to 1.20% in the pandemic report by Oette et al. [99], while 9 out of 1273 samples were positive for influenza (0.71%), and 752 out of 7375 were positive for SARS-CoV-2 (10.20%).

**Figure 2:**
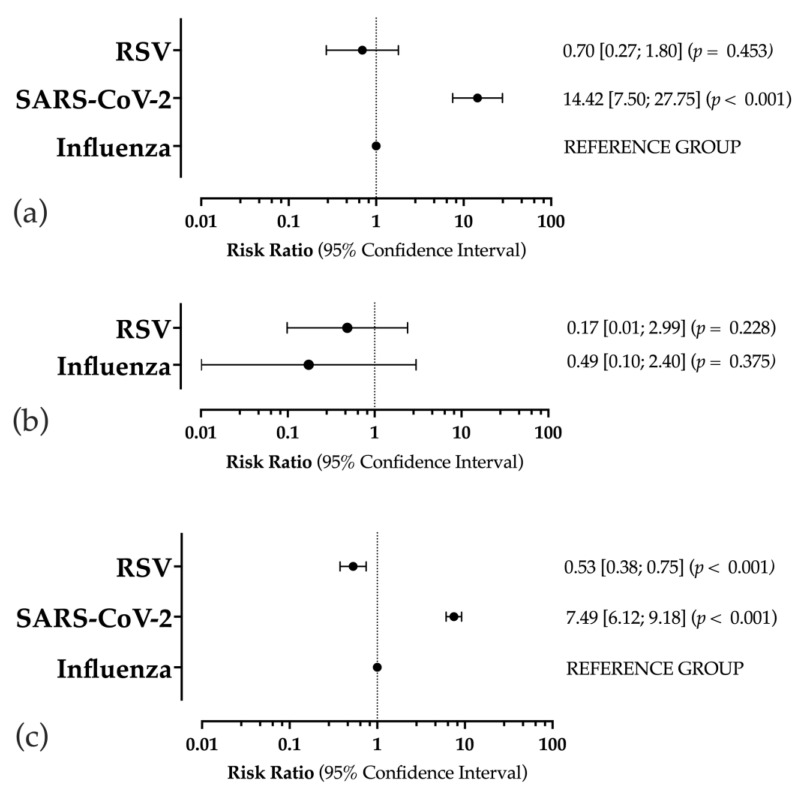
Forest plot reporting Risk Ratios (RRs) with their corresponding 95% confidence intervals (95%CI) for the occurrence of positive cases among the sampled homeless people: subfigure (**a**), prevalence estimates, whole of assessed timeframe; subfigure (**b**): prevalence estimates, post-pandemic studies (data retrieved starting with January 2020) are compared to pre-pandemic studies (data collected before January 2020) [4,5,6,17,18,42,98,99,100,101,102,103,104,105,106,107,108]; subfigure (**c**): positive samples from incidence studies, positive rates for influenza are considered the reference group [19,20,21,44,109,110,111,112,113,114,115,116].

As shown in Figure 2a, when the cumulative prevalence for influenza was considered the reference group, no substantial differences were identified for the RSV prevalence (RR: 0.70; 95%CI: 0.27 to 1.80; *p* = 0.453). On the contrary, an increased occurrence of SARS-CoV-2 (RR: 14.42; 95%CI: 7.50 to 27.75; *p* < 0.001) was documented.

Interestingly (Figure 2b), no differences in the prevalence estimates were reported between the pre- and post-pandemic studies for RSV and influenza.

### 3.6. Incidence Estimates

Overall, the highest cumulative occurrence (i.e., the number of positive tests over the total number of collected specimens) was calculated for SARS-CoV-2 (4.69%) (Table 5). Regarding RSV, the positive proportion ranged between 0.14% and 1.82%, with a cumulative occurrence of 0.30%, compared to the cumulative occurrence of 0.56% for influenza. The crude incidence rates were 1.74 per 1000 person-months for RSV (95%CI: 0.00 to 7.94), 6.40 per 1000 person-months for influenza (95%CI: 0.00 to 21.58) and 8.73 per 1000 person-months for SARS-CoV-2 (95%CI: 0.96 to 16.49).

As shown in Figure 2c, when influenza was taken into account as the reference group, the occurrence of positive samples was significantly lower for RSV (RR: 0.53; 95%CI: 0.38 to 0.75; *p* < 0.001), while significantly higher estimates were associated with SARS-CoV-2 (RR: 7.49; 95%CI: 6.12 to 9.18; *p* < 0.001).

### 3.7. Outcome Estimates

Overall, 64 cases of RSV infections (0.26% of sampled HP), 148 cases of influenza infections (0.59%) and 466 cases of SARS-CoV-2 infections (4.76%) were included. As the study by Boonyaratanakornkit et al., 2019 [117] only reported hospitalization rates, the estimates for both RSV and influenza are reasonably underestimated. When compared with the reference non-HP population, both pathogens were associated with increased odds of hospitalization (OR: 6.71 and 95%CI: 4.79 to 9.38 for RSV; OR: 3.45 and 95%CI: 2.86 to 4.16 for influenza), even though Rogers et al. [19] hint at no substantial differences in healthcare assistance requests due to RSV compared to influenza (OR: 1.18; 95%CI: 0.21 to 7.50). Similarly, the HP in the study by Richard et al. [112] were associated with a substantially increased frequency of hospitalization due to SARS-CoV-2 compared to the reference population (1.23% vs. 0.29%; OR: 4.11; 95%CI: 3.21 to 5.26), while ICU admissions (OR: 1.51; 95%CI: 0.89 to 2.51) and deaths (OR: 1.44; 95%CI: 0.75 to 2.68) were similarly reported among the non-HP population and HP population. On the contrary, according to Boonyaratanakornkit et al. [117], the proportion of ICU admissions among RSV cases was higher than among cases of influenza (25% vs. 17%; *p* = 0.041), as were the 30-day readmission rates (25% vs. 11%).

### 3.8. Risk of Bias

**Figure 3:**
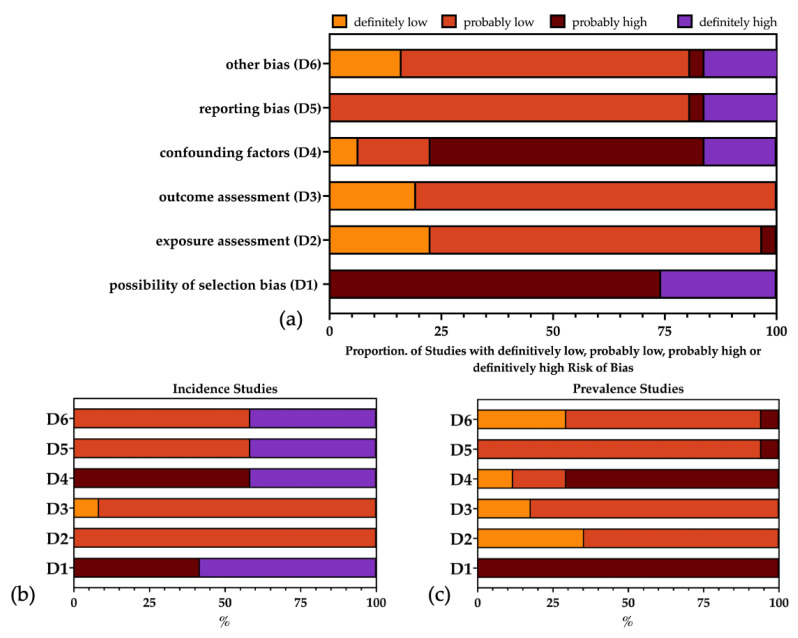
Summary of the risk of bias (ROB) estimates for the observational studies [89,119]. Analyses were performed according to the National Toxicology Program’s (NTP) Office of Health Assessment and Translation (OHAT) handbook and respective risk of bias (ROB), tool including all the retrieved studies (N. = 31, (**a**)), and the settings of the studies, i.e., prevalence studies (N. = 17, (**b**)) [4,5,6,17,18,42,98,99,100,101,102,103,104,105,106,107,108] and incidence studies (N. = 12; (**c**)) [19,20,21,44,109,110,111,112,113,114,115,116].

The risk of bias (ROB) assessment of the retrieved studies is analytically reported in Table 6 and summarized in Figure 3.

Even though the overall quality of the collected sample was relatively high, all the studies, particularly the reports from the Seattle area, were affected by significant selection bias (D1), as it is unclear how participating HP differed from non-participating ones. As a large proportion of the studies contributing to the incidence estimates were retrieved from the studies performed in the Seattle area, the risk of bias in D1 was particularly high in this subgroup. An exposure assessment (D2) and outcome assessment (D3) were properly reported by all studies, while the lack of detailed demographics and personal risk factors resulted in the inappropriate appraisal of potential confounding factors (D4), particularly in the incidence studies. Regarding potential reporting bias (D5), the studies from France were either reasonably not affected or only limitedly affected, as they included the individual characteristics of the participating HP, contributing to a better appraisal of the prevalence studies compared to the incidence studies. In fact, all of the American studies were not only affected by some degree of reporting bias but also by some inaccuracies in the description of the sample (D6) because of their design, the overlap of participating HP and the repeated sampling of the same participants, which was not consistently stated across the various studies.

### 3.9. Meta-Analysis

#### 3.9.1. Prevalence Estimates

**Figure A1:**
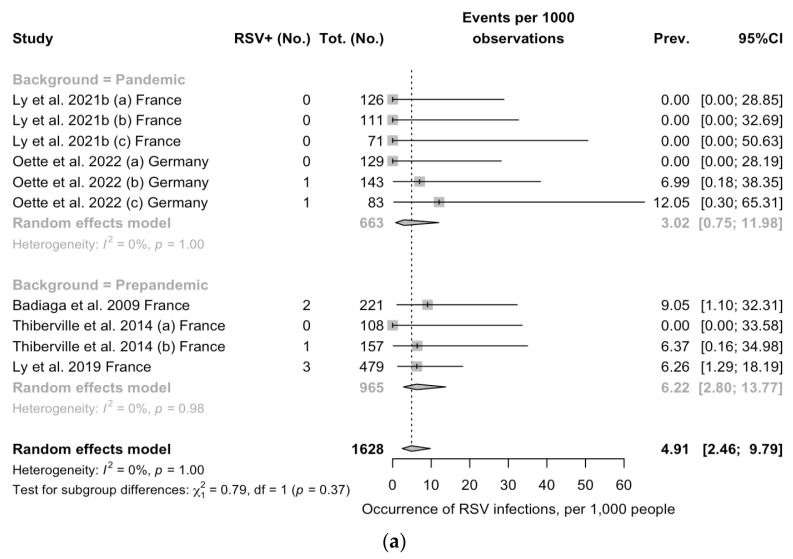

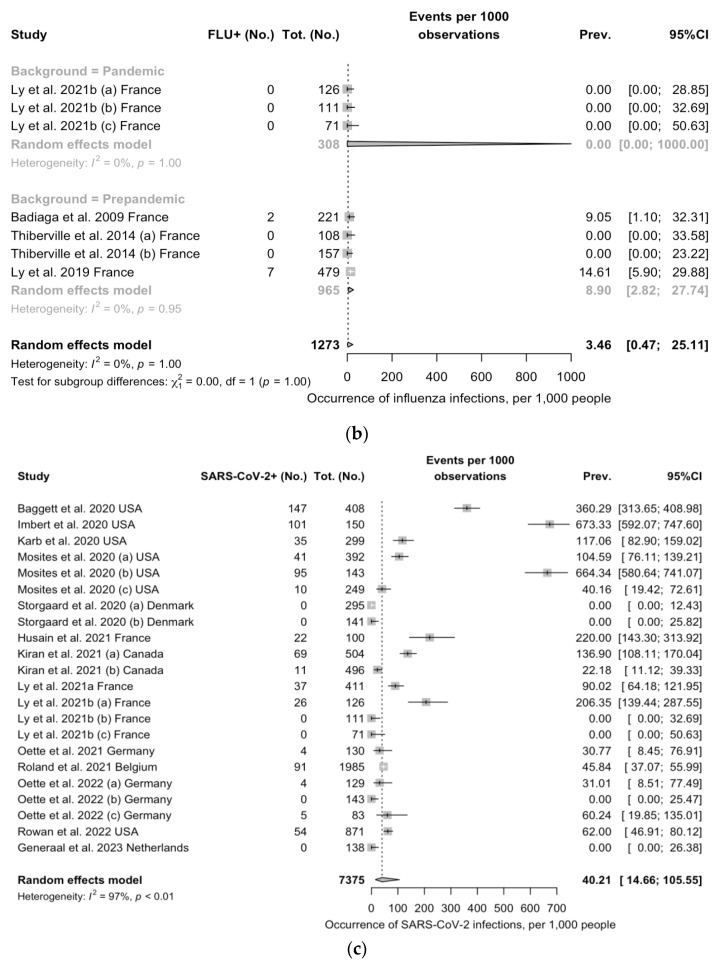
Forest plot for prevalence studies on RSV (**a**), influenza (**b**) and SARS-CoV-2 infections (**c**) among homeless people. All estimates are reported in cases per 1000 people [4,5,6,17,18,42,98,99,100,101,102,103,104,105,106,107,108].

The pooled prevalence rates (reported episode per 1000 people) were estimated through an REM meta-analysis, and the corresponding estimates are shown in Table 7, while the individual estimates are reported in Appendix A, Figure A1. Overall, a pooled prevalence rate of 4.91 per 1000 people (95%CI: 2.46 to 9.80) was calculated for RSV, compared to 3.47 per 1000 people (95%CI: 0.47 to 25.11) for influenza and 40.21 per 1000 people (95%CI: 14.66 to 105.55) for SARS-CoV-2.

The residual heterogeneity was seemingly low for RSV and influenza and substantial for SARS-CoV-2 (I^2^ = 97.5%). However, the corresponding 95%CIs suggest a quite different pattern. In fact, the upper limits of the I^2^ estimates for RSV (62.4%) and influenza (70.8%) exceeded the cut-off of 50.0% for substantial heterogeneity, which, because of the reduced number of sampled studies, cannot therefore be ruled out.

**Figure 4:**
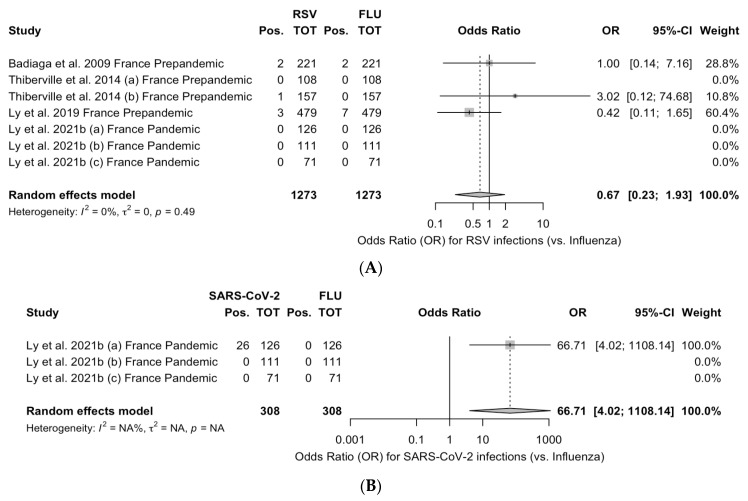
Forest plots reporting the odds of developing RSV (**A**) and SARS-CoV-2 infections (**B**) among homeless people compared to influenza in the same studies [4,5,6,17,18,42,98,99,100,101,102,103,104,105,106,107,108] (Distinctive series within the same study are noted with progressive letter).

Taking the prevalence of influenza as the reference group, the pooled ORs for RSV and SARS-CoV-2 were calculated, and the corresponding estimates are reported in Figure 4. When dealing with pooled estimates, it should be stressed that the three series on SARS-CoV-2 were drawn from the study by Ly et al., 2021 [5] and are therefore limitedly comparable to the estimates for RSV, which encompassed seven series from three different studies [4,5,17,18]. Even though the I^2^ estimate for RSV was below 60%, the corresponding upper limits of the 95%CI exceeded the cut-off for substantial heterogeneity (95%CI: 0.0 to 89.6), suggesting a cautious appraisal of the included data.

#### 3.9.2. Incidence Estimates 

**Figure A2:**
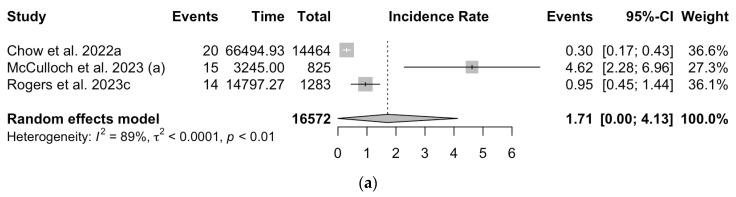

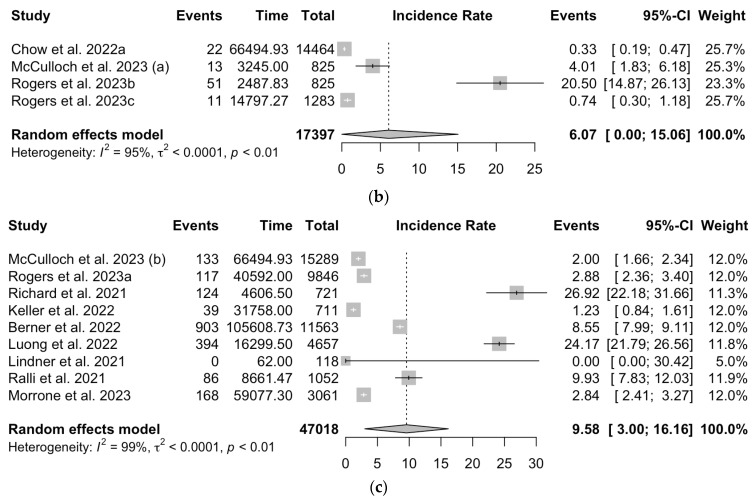
Forest plot for incidence studies on RSV (**a**), influenza (**b**) and SARS-CoV-2 infections (**c**) among homeless people. All estimates are reported in cases per 1000 person-months [19,20,21,44,109,110,111,112,113,114,115,116].

A summary of the incidence estimates is provided in Table 8, while the corresponding forest plots are provided in Appendix A, Figure A2.

As shown, SARS-CoV-2 was associated with the highest estimate of 9.58 per 1000 person-days (95%CI: 3.00 to 16.16), followed by influenza (6.07, 95%CI: 0.00 to 15.06), while RSV was associated with the lowest ones (1.71, 95%CI: 0.00 to 4.13). All the estimates were affected by substantial heterogeneity (I^2^ > 50.0%).

Because of the limited number of studies and retrieved series, no pooled analysis of the outcome studies was ultimately carried out.

### 3.10. Sensitivity Analysis

**Figure A3:**
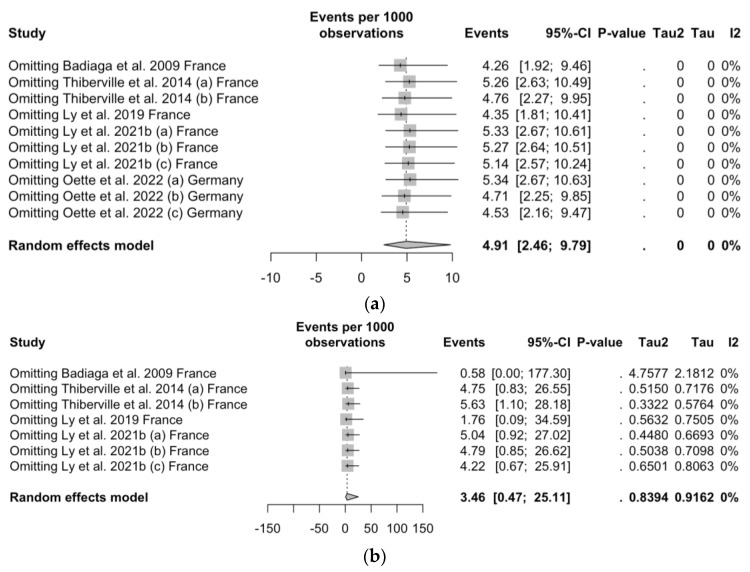

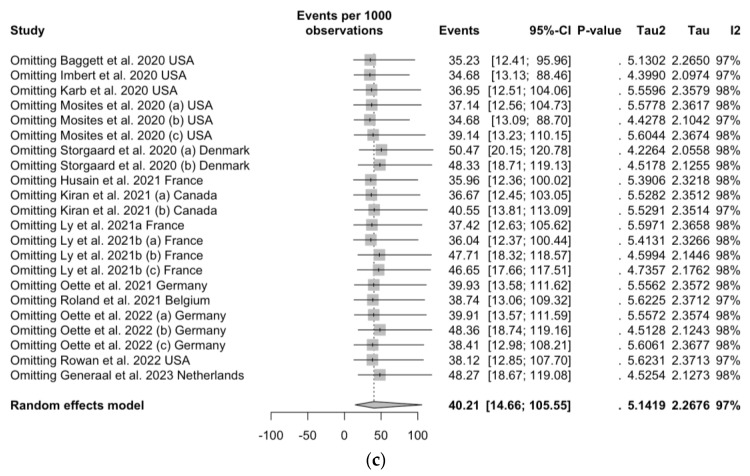
Sensitivity analysis on prevalence studies on RSV (**a**), influenza (**b**) and SARS-CoV-2 (**c**) in homeless people. Analyses were performed through the approach of removing a single study at a time [4,5,6,17,18,42,98,99,100,101,102,103,104,105,106,107,108] (Distinctive series within the same study are noted with progressive letter).

**Figure A4:**
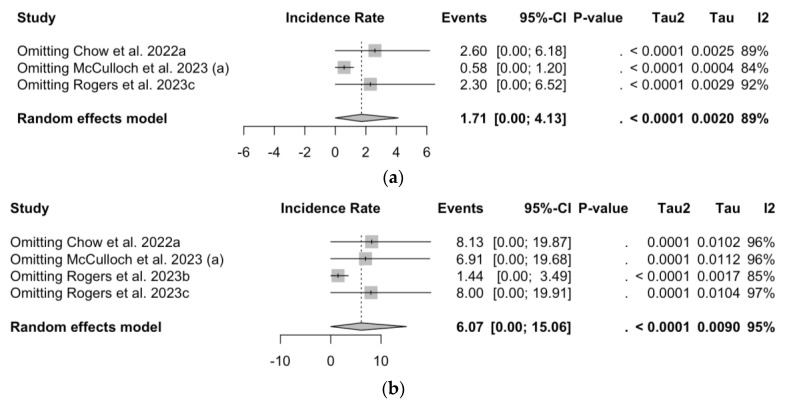

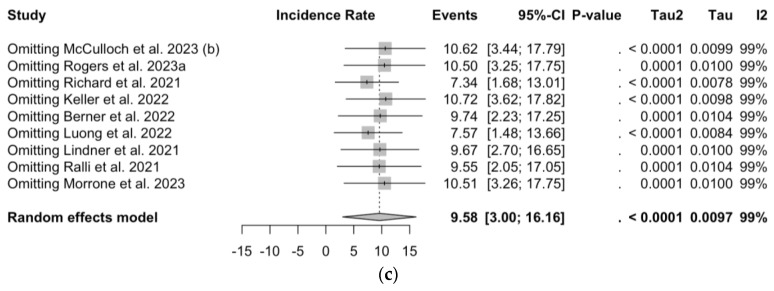
Sensitivity analysis on incidence studies on RSV (**a**), influenza (**b**) and SARS-CoV-2 (**c**) in homeless people. Analyses were performed through the approach of removing a single study at a time [19,20,21,44,109,110,111,112,113,114,115,116].

The sensitivity analysis was performed by removing a single study at a time, and the pooled estimates are reported in Appendix A, Figure A3 for the prevalence studies and in Appendix A, Figure A4 for the incidence studies. Regarding the prevalence studies, the removal of a single study at a time did not affect the pooled estimates of the residual heterogeneity for RSV and influenza, with point estimates consistently remaining unnoticeable. Similarly, the removal of the series on SARS-CoV-2 did not affect or reduce the I^2^ estimates, which consistently remained >95%.

The sensitivity analysis of the incidence studies led to similar results, as the I^2^ point values remained substantially high in all the estimates. Still, it can be noticed that the removal of the study by Rogers et al. [44] from the RSV and influenza estimates reduced the residual heterogeneity, which decreased from 89–92% for RSV to 84% and from 96–97% for influenza to 85%, with pooled estimates decreasing to, respectively, 0.58 RSV cases per 1000 persons-month (95%CI: 0.00 to 1.20) and 1.44 influenza cases per 1000 persons-month (95%CI: 0.00 to 3.49).

### 3.11. Analysis of Publication Bias and Small-Study Bias

**Figure A5:**
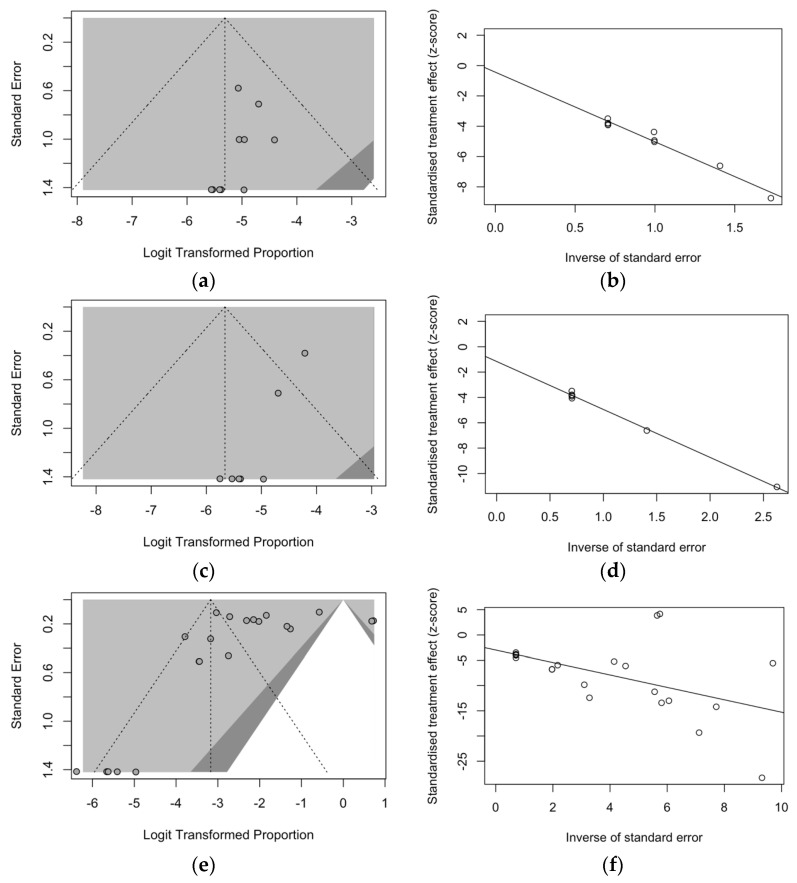
Funnel plots for studies on prevalence rates for respiratory pathogens included in the analyses, (**a**) respiratory syncytial virus (RSV); (**c**) influenza; (**e**) SARS-CoV-2, and corresponding radial plots (RSV, (**b**); influenza, (**d**), SARS-CoV-2, (**f**) [4,5,6,17,18,42,98,99,100,101,102,103,104,105,106,107,108].

**Figure A6:**
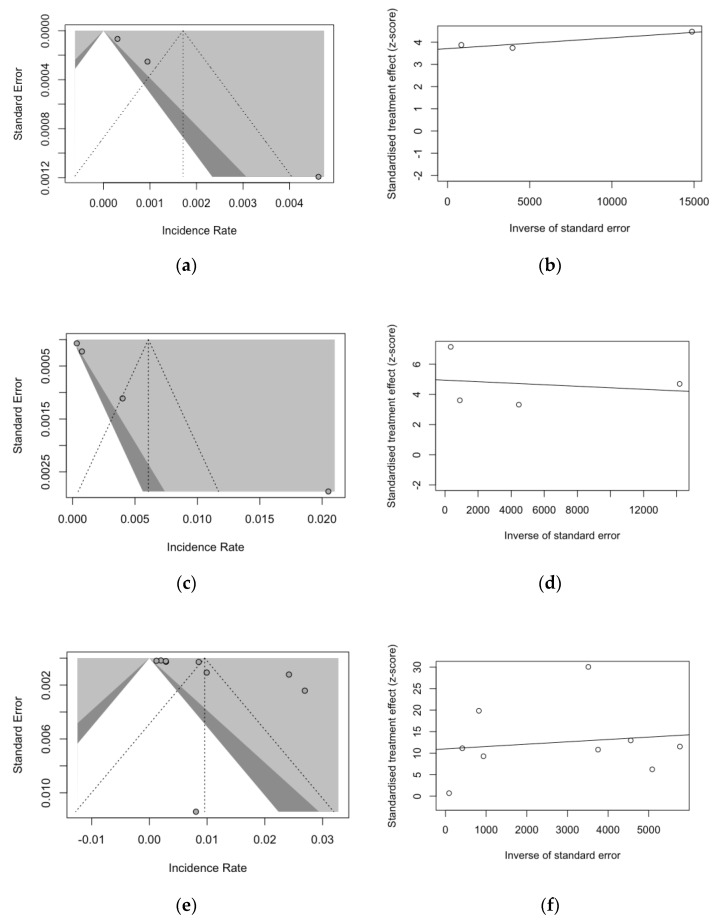
Funnel plots for studies on incidence rates for respiratory pathogens included in the analyses, (**a**) respiratory syncytial virus (RSV); (**c**) influenza; (**e**) SARS-CoV-2, and corresponding radial plots (RSV, (**b**); influenza, (**d**), SARS-CoV-2, (**f**) [19,20,21,44,109,110,111,112,113,114,115,116].

The publication bias was initially ascertained through the calculation of funnel plots. In funnel plots, the sample size is plotted against the effect size they report. As the size of the sample increases, the individual estimates of the effect are likely to converge around the true underlying estimate [63,66,73]. The funnel plots of the prevalence rates are reported in Appendix A, Figure A5a,c,e. All the funnel plots were substantially asymmetrical, as the points predominantly pointed towards the right, with nearly half of the estimates clustered in the lower half of the plot, suggesting the presence of publication bias with a high share of lower-precision studies. In other terms, the meta-analysis summary possibly underestimated the prevalence rates for respiratory pathogens, leading to a reasonable bias due to the small size of the studies. Taking into account that the pooled analyses on the incidence rates are affected by the very limited number of collected series and studies, requiring a more cautious appraisal of the visual inspections of funnel plots, similar considerations can be shared for the estimates of RSV, influenza and SARS-CoV-2 incidence (Appendix A, Figure A6a,c,d).

Radial plots were similarly calculated and are reported in Appendix A, Figure A5b,d,e for prevalence rates, and Appendix A, Figure A6b,d,e for incidence rates. The point estimates of the prevalence of RSV and influenza were seemingly scattered across the upper and lower sides of the regression line, while the estimates of the prevalence of SARS-CoV-2 appeared more clearly scattered around the lower side of the regression line (Appendix A, Figure A6f). Such findings were only partially confirmed by Egger’s test (Table 9), which hinted towards a substantial publication bias for the studies on influenza alone (t = −8.46, *p* < 0.001). In turn, the radial plots for the incidence studies were seemingly spared by the clustering of retrieved data, but the results of Egger’s test stress a likely publication bias for all of the estimates (in all cases, *p* < 0.100).

## 4. Discussion

### 4.1. Key Findings

In our systematic review and meta-analysis, we conveyed and summarized evidence from 31 studies dealing with the occurrence and outcome of RSV, influenza and/or SARS-CoV-2 infections in HP.

A total of 17 prevalence studies were retrieved [4,5,6,17,18,42,98,99,100,101,102,103,104,105,106,107,108], with resulting pooled estimates of 4.91 cases per 1000 HP (95%CI: 2.46 to 9.80) for RSV, 3.47 per 1000 HP for influenza and 40.21 cases per 1000 HP (95%CI: 14.66 to 105.55) for SARS-CoV-2. The meta-analysis results suggest that the mitigation strategies enforced since the inception of the SARS-CoV-2 pandemic [19,102,120,121,122] may have reduced the circulation of RSV and influenza, as in both cases, the prevalence estimates exhibited a significant decrease, particularly for influenza (from 8.90 per 1000 HP, 95%CI: 2.82 to 27.74, to no case detected), and more limitedly for RSV (from 6.22 per 1000 HP, 95%CI: 2.80 to 13.77, to 3.02 per 1000 HP), hinting at a residual circulation of that pathogen. The estimates for RSV and influenza were seemingly less affected by heterogeneity issues than those for SARS-CoV-2, but this was somewhat expected, as a large share of the samples were either retrieved from studies performed in the same areas, even in the same shelters, or that shared the same blueprint [98,99,100]. Interestingly enough, the meta-analysis results appear to somewhat conflict with the crude estimates, which documented no significant difference between the pre-pandemic and pandemic timeframes (RR: 0.17; 95%CI: 0.01 to 2.99 for RSV and RR: 0.49; 95%CI: 0.10 to 2.40 for influenza). As these differences could be due to the small number of studies retrieved and the heterogenous sample size, our results should be analyzed with care.

The incidence rates were calculated from 12 studies [19,20,21,44,109,110,111,112,113,114,115,116], mostly from the United States [19,20,21,44,113,114,115] and Canada [112,116], and again, SARS-CoV-2 was characterized by the highest occurrence (9.58 diagnoses per 1000 persons-month, 95%CI: 3.00 to 16.16), followed by influenza (6.07, 95%CI: 0.00 to 15.06) and RSV (1.71, 95%CI: 0.00 to 4.13). The reliability of the aforementioned estimates was limited, particularly when dealing with RSV (only 3 series and 49 events) and influenza (4 series and 97 events), as stressed by the calculation of the residual heterogeneity, the sensitivity analysis and the analysis of the publication bias. In analogy with the prevalence studies, the pooled estimates included HP recruited from the same parent population (in this case, Seattle and King’s County), but while the former studies replicated a common design without overlapping in terms of the assessed timeframe, the latter are affected by substantial overlaps in the observation period and coexisting differences in the reporting strategy. Moreover, some of the incidence studies reported on both the pandemic and pre-pandemic timeframes. For instance, Chow et al. [21], as well as one of the series from the report by McCulloch et al. [20], included data from October 2019 to May 2021, while Rogers et al. [44] reported on the whole of the winter season in 2019–2020. Nonetheless, even reports more clearly focused on either the pre-pandemic or pandemic timeframe, such as those by Ralli et al. [109], Lindner et al. [111], Morrone et al. [110] and Rogers et al. [114], were affected by the various and heterogenous timing of the removal of physical distancing and lockdown, which influenced the circulation of respiratory pathogens, as well as by the emergence of new and more infectious variants of SARS-CoV-2 [123,124].

As an even more limited number of studies provided the outcome estimates, a pooled quantitative analysis was not performed. However, the retrieved data hint at increased odds of hospitalization due to RSV (OR: 6.71; 95%CI: 4.79 to 9.38), influenza (OR: 3.45; 95%CI: 2.86 to 4.16) and RSV compared to the reference population (1.23% vs. 0.29%; OR: 4.11; 95%CI: 3.21 to 5.26). Interestingly, the frequency of requests for healthcare assistance was similar in RSV cases and influenza infections (OR: 1.18; 95%CI: 0.21 to 7.50). Moreover, ICU admissions (OR: 1.51; 95%CI: 0.89 to 2.51) and deaths (OR: 1.44; 95%CI: 0.75 to 2.68) due to SARS-CoV-2 were similarly reported among the non-HP population and the HP population, suggesting that factors other than the severity of the infection may be associated with the increased hospitalization rates.

### 4.2. Generalizability

A key issue when dealing with studies on HP is represented by the high variability in baseline characteristics depending on the country and the type of study [125], and even within the same country, demographic characteristics are highly variable and heterogenous [12,42,126]. For example, a previous report by Hwang [126] stressed that around 2000 families with children occupied up to 42% and 35% of shelters in Toronto and Ottawa, respectively. On the contrary, in other Canadian cities and main centers, single men represented a large majority of sheltered HP. Therefore, the collected estimates should only be cautiously generalized, being strictly dependent on the specific area where the study was performed [12,42,125,126], as otherwise stressed by the substantial heterogeneities arising from the pooled estimates. As the available data were mainly collected from two areas (i.e., Marseille and the state of Washington), the limited reliability from a global health perspective cannot be underestimated. Moreover, we deliberately focused on HP recruited from urban shelters, while a large majority of HP are represented by refugees and displaced people either living on the streets, in camps and/or in specifically designed shelters [96,127,128,129]. In this regard, it is important to stress that we deliberately excluded data on refugees from the present analysis. Indeed, this specific subgroup is usually characterized by a high proportion of individuals of the female gender and either low or high age groups, which is therefore quite inconsistent with the usual description of HP from urban shelters in high-income countries. In fact, in our study, a large majority of the sampled HP were of the male gender, with either a mean or median age of around 40 years. On the contrary, in a recent report by Siddik et al. [127], female refugees accounted for 46% of the participants, and 59% of the participants were less than 5 years old at the time of the analyses. Similarly, in a previous report by Ahmed et al. on refugee camps in Kenya, female subjects accounted for 45.8% of the sampled individuals, and only 18.0% of the 6264 specimens were collected in individuals aged more than 5 years [129].

On the other hand, some common features of HP have been widely acknowledged and should be considered. Among HP, the prevalence of alcohol abuse, IV drug abuse and heavy tobacco smoke is significantly higher than in the general population; for instance, in an American study by Segal SP et al. [130], up to 78% of the patients were heavy smokers, 50% were affected by IV drug abuse and 21% were addicted to alcohol. Although accurate reporting on demographics and risk factors was not consistently available for all the retrieved studies, in our pooled sample, we identified high rates of smoking habits as well as alcohol and substance abuse. Substance abuse is particularly significant for the aims of the present review, as it is associated with weakened immunity and other biological abnormalities that predispose people to specific infections and eventually to higher mortality rates. For example, among male HP sheltered in Toronto, the mortality rates were found to be 8.3 times higher than the mean mortality for 18–24-year-olds, 3.7 times higher than the mean for 25–44-year-olds and 2.3 times higher than the mean for 45–64-year-olds [13,42,126,131,132]. Among the main causes of morbidity and mortality for HP, respiratory disorders, including bronchitis, chronic coughs and pulmonary infections, are frequently reported [12,125,126]. Not only are minor upper respiratory infections twice as common in homeless children compared to the general population [5,9,17,19,20] but hospitalizations and deaths due to respiratory diseases are also more frequently reported among HP than among the general population [9,17,125], particularly in men rather than in women [12]. For example, between 1988 and 1993, respiratory infections caused up to 20% of the total deaths in the Boston homeless population [131,132], and even in a recent report by Romaszko et al. [7], including a total of 142 deaths among HP from Poland, 16 events (11.27%) were associated with diseases of the respiratory system, with a strong association with cold environmental temperatures (*p* < 0.001; W: 11.76). However, the outcome studies suggested that while HP may be affected by higher rates of hospital admissions due to respiratory pathogens than the general population, ICU admission and lethality were not significantly different from those reported in the reference groups [117,118]. A likely explanation could be that while respiratory syndrome associated with milder to moderate symptoms can usually be managed in home settings, hospitalization may represent the only available option for guaranteeing appropriate management in HP.

Homeless shelters represent suitable settings for the transmission of respiratory pathogens, as previously proven by studies on bacterial pathogens, particularly *Mycobacterium tuberculosis* [22,23,24,25]. In a study from Australia, molecular analyses of 19 isolates of *M tuberculosis* from HP showed that 18 had the same genetic profile, proving the occurrence of people-to-people transmission within the same shelter [133]. A subsequent study based in the Paris area suggested that HP may easily act as vectors for the same strain of *M tuberculosis* across various shelters, as 35% of the 177 strains isolated in Paris were found in 26 groups of 2 to 12 individuals highly associated with homelessness [134]. In shelters, contagious diseases can easily spread by aerosols as well as by direct contact, and the close proximity in crowded shelters provides the ideal conditions for the spreading of respiratory infections, including viral ones such as influenza [4,5,17] and SARS-CoV-2 [6,41,42,135]. For example, in a recent study by Roederer et al. [136], crowded living conditions (e.g., sharing a room with more than five people and sharing a bathroom with more than five people) were associated with higher odds for developing a SARS-CoV-2 positive status (OR: 4.3, 95%CI: 2.2–8.4 and OR: 3.1, 95%CI: 2.0–5.0, respectively). Even when adjusted for gender, the frequency of leaving the place of residence, the crowding in the place of residence, tobacco consumption and recruitment sites, the odds for seropositivity increased with crowding (aOR: 2.7; 95%CI: 1.5 to 5.1). Even in our study, the incidence for SARS-CoV-2 infections was disproportionately higher than that reported for RSV and influenza, although these figures were reasonably inflated by the oversampling of HP during the early stages of the pandemic. The retrieved reports stress how rapidly a SARS-CoV-2 outbreak could spread across the homeless shelters and the need for raising and maintaining appropriate preventive and containment measures even in post-pandemic settings—for example, by achieving and maintaining high vaccination rates [137,138].

The circulation of RSV among the sampled HP and the corresponding prevalence and incidence rates were more limited but still noticeable, even during the pandemic timeframe, and these results were not unexpected, retaining substantial significance from a public health point of view. Contrarily to common stereotypes, the burden of disease associated with RSV infections in adults and the elderly is far from negligible [70,139,140]. In 2005, Falsey et al. [70] estimated an incidence of RSV infection in older adults (timeframe: 1999 to 2003) ranging between 3% and 7% based on RT-qPCR and serology, while a similarly designed European study has suggested an incidence ranging between 4.2% and 7.2% [141,142]. The available reports suggest that RSV in adults may be associated with an unexpectedly high rate of complications. Oxygen therapy was more commonly administered to patients hospitalized due to RSV rather than those hospitalized due to COVID-19 or influenza (aOR: 2.97; 95%CI: 2.07–4.27 for COVID-19; aOR: 2.07; 95%CI: 1.37–3.11 for influenza), as was non-invasive ventilation (aOR: 2.25; 95%CI: 1.65–3.07 for COVID-19 and aOR: 1.99; 95%CI: 1.36–2.90 for flu) and admittance to an ICU (aOR: 1.49; 95%CI: 1.13–1.97 for COVID-19 and aOR: 1.55; 95%CI: 1.11–2.19 for influenza) [71]. In this regard, a recent study on 25 hospitals in France (time span: from 1 January 2015 to 31 December 2019) on a total of 1168 adults and elderly people hospitalized for RSV infections demonstrated that about one-fourth of them required ICU admission [139]. Interestingly enough, obesity (aOR: 1.78; 95%CI: 1.26 to 2.53), hypertension (aOR: 1.45; 95%CI: 1.05 to 1.99), chronic heart failure (aOR: 2.18; 95%CI: 1.56 to 3.03), COPD (aOR: 2.79; 95%CI: 1.90 to 4.09) and chronic respiratory failure (aOR: 1.64; 95%CI: 1.10 to 2.44) were associated with an increased risk of ICU admission [139], and all of the aforementioned conditions are more frequently reported among HP than in the general population [7,13,126,131,132,143]. Moreover, these conditions frequently overlap; in a study by LaWall et al. [144], the prevalence of individuals reporting ≥ four comorbidities was 76%, compared to 62% in the general population. Furthermore, HP tend not to refer to the regular healthcare system when they become sick, and even when treated, their adherence is often poor [145] for a series of different reasons: not only because they are often unable to pay for their treatment [17,125,126], but also because of underlying factors such as mental illness, transport problems, self-neglect and fear of institutions [7,9,22,28,125,126]. Again, these features highlight that hospitalization could represent the only available option for treating HP affected by mild-to-moderate respiratory infections, leading to a likely overestimation of the corresponding rates.

When dealing with our estimates of incidence rates, a particularly cautious appraisal is suggested according to the known epidemiology of RSV. On the one hand, RSV, particularly before the SARS-CoV-2 pandemic, was characterized by a well-defined seasonal trend [46,48,61]. As a consequence, reports stretching across the whole of the calendar year could reasonably underestimate the actual occurrence of this respiratory infection, while reports focused on the winter season alone could conversely provide significant overestimations. On the other hand, the actual incidence rate in the general population of RSV infection is usually considered unclear because of the high proportion of cases without a laboratory diagnosis, and it is highly variable within and across the seasons and calendar years [61,146,147,148,149]. Therefore, an appropriate analysis of the retrieved data would require a comparison with background circulation estimates of RSV infection, but unfortunately, these data are not extensively available. Nonetheless, taking into account the documented circulation of RSV even when influenza was substantially undetectable among the sampled HP, the potential severity of RSV infections among the HP and the pooled occurrence estimates for RSV infections we identified [74,150], it is reasonable that HP could benefit from preventive interventions such as those implemented for influenza and COVID-19 [74,150], including vaccination. Not only has the SARS-CoV-2 vaccine been recommended to HP since the inception of the vaccination campaign by several public health authorities [9,137,151], but some recommendations for the seasonal influenza vaccine have been recently issued. For example, the Australian Department of Health and Aged Care has recently recommended that homeless people receive an annual influenza vaccine [152]. On the contrary, the use of RSV vaccines among HP remains an uncharted territory. After decades of intensive research, two RSV vaccines have been recently approved (RSVPreF3, commercial name Arexvy^®^ from GSK, and RSVPreF, commercial name Abrysvo^®^ from Pfizer) [71]. Both can be delivered as a single dose and have been proven to be safe and quite efficient among adults in preventing RSV infections and their severe complications [76,153,154,155]. So far, RSV vaccines appear to provide at least partial protection for a minimum of two RSV seasons [71,76,153,154,155], and their delivery could therefore be quite useful in preventing RSV infections in homeless settings. Unfortunately, both formulations have been licensed only to individuals aged ≥ 60 years, with some indications for pregnant women [80,81]. Not only did elderly people represent a very limited subgroup of the sampled HP, questioning the cost-effectiveness of this potential intervention, but when demographic data were provided, outcome estimates by age groups were not available. For example, in the reports from Oette et al. [99,100], the age group 60 years and older encompassed between 13.5% and 10.8% of the sampled individuals, but no information was then provided about the occurrence of respiratory infections, including RSV, by age group. Consequently, the potentially dismal outcome of RSV infections in older HP could be only hinted at by the general outcome data on RSV infections in HP and in elderly people from the general population, but it is not directly proven. RSV vaccination campaigns among HP from shelters could therefore be suggested as a preventive measure for people who more reluctantly benefit from healthcare assistance in earlier stages of respiratory infections rather than for reducing the occurrence of this pathogen and improving its outcomes in the enclosed and high-risk settings of homeless shelters.

### 4.3. Limits and Implications for Future Studies

Despite its potential significance from a public health point of view, our study is affected by several significant limits that should be considered.

First, although the overall assessment of the included reports suggested appropriate or even relatively high quality (particularly for the prevalence studies from Marseille and Paris shelters) [4,5,17,18], most of the RSV and influenza samples that were collected in the USA were affected by potential issues when dealing with the selection of the study population and the reporting of potential risk factors. Actually, individual features of HP have been often described as strikingly heterogeneous across the various settings, not only regarding their baseline demographics but also when dealing with underlying risk factors (e.g., the proportion and severity of substance abuse, potential reliance on healthcare interventions from local health and governmental authorities, etc.) [2,9,16,19,22,25,125,126]. Unfortunately, these data were irregularly provided, and our overall understanding of the global epidemiology of respiratory diseases among HP could therefore have been flawed by not considering risk factors unrelated to living in homeless shelters.

Second, as several of the studies were performed in the very same areas, we are substantially unable to identify the proportion of patients that were repetitively sampled between 2009 and 2022, particularly when dealing with estimates from the Marseille area [4,5,17,18]. Our pooled sample may therefore be affected by the extensive oversampling and the duplicate sampling of certain individuals, characterized by higher attention being paid to their health features and a higher sensitivity towards potential infectious diseases. As a consequence, our estimates can only be seen as a proxy for the actual epidemiology of respiratory pathogens among HP from shelters. In fact, according to the reports, the individual series included 20.83% to 44.52% of the potentially targeted population [4,5,17,18]. As all the studies were based on the voluntary participation of the potentially sampled HP, all the studies were reasonably affected by some degree of self-selection of participants, with a likely overrepresentation of individuals characterized by a higher trust in healthcare authorities and providers. As commonly acknowledged, HP subgroups such as people affected by mental illnesses and IV drug addiction are often very cautious in their interactions with governmental bodies [2,7,9,15,18,22,27,126,131,132], despite the fact that these subgroups would benefit the most from healthcare [13,42,125,126,131,132].

Third, as our study only included HP from urban shelters, it was limitedly representative of the whole of the urban HP population, as sheltered HP only represent a fraction of the four main categories of homelessness according to the European Typology of Homelessness and Housing Exclusion (ETHOS) [8,41], which also include people living roofless, houseless and in settings of insecure and/or inadequate housing. As stressed by Hwang more than 20 years ago [12,126], unsheltered HP in their forties and fifties often develop health disabilities that are more commonly seen in people who are decades older, and these features may possibly lead to a dismal prognosis of respiratory tract infections. These patients could therefore benefit from interventions otherwise associated with older age groups, not only including the potential delivery of RSV vaccines, but also by prioritizing the use of higher-dose flu vaccines, whose efficacy in older subjects has been otherwise proven [156,157,158,159].

Therefore, future studies are highly needed for a more accurate appraisal of the actual epidemiology of viral respiratory pathogens in urban homeless shelters, not only in high-income settings but also in middle- and low-income countries [47,48,61,160].

## 5. Conclusions

RSV, influenza and SARS-CoV-2 infections in HP from urban homeless shelters are a likely occurrence, and even though the occurrence of RSV and influenza declined during the SARS-CoV-2 pandemic, their circulation remained noticeable even when lockdown measures were globally implemented. In this regard, non-pharmaceutical interventions were limitedly effective in avoiding SARS-CoV-2 outbreaks in the enclosed settings of HP urban shelters. High rates of hospitalization, ICU admission and even death were documented, although more limitedly. Because of the specific features of homelessness, the availability of effective immunizations against these pathogens stresses the potential public health and social value of RSV, influenza and SARS-CoV-2 vaccines in this subgroup of the adult population.

## Figures and Tables

**Table 4:** Occurrence of respiratory viruses in collected specimens from prevalence studies.

Study	Total Sample (N./8340., %)	RSV (n./N, %)	Influenza (n./N, %)	SARS-CoV-2 (n./N, %)
Badiaga et al., 2009 [17]	221 (2.65%)	2 (0.90%)	2, 0.90%	-
Thiberville et al., 2014 [18]	108 (1.29%)	0 (-)	0 (-)	-
	157 (1.88%)	1 (0.64%)	0 (-)	-
Ly et al., 2019 [4]	479 (5.74%)	3 (0.63%)	7 (1.46%)	-
Baggett et al., 2020 [108]	408 (4.89%)	-	-	147 (36.03%)
Imbert et al., 2020 [107]	150 (1.80%)	-	-	101 (67.3%)
Karb et al., 2020 [106]	299 (3.59%)	-	-	35 (11.71%)
Mosites et al., 2020 [105]	392 (4.70%)	-	-	41 (10.46%)
	143 (1.71%)	-	-	95 (66.43%)
	249 (2.99%)	-	-	10 (4.02%)
Storgaard et al., 2020 [104]	295 (3.54%)	-	-	0 (-)
	141 (1.69%)	-	-	0 (-)
Husain et al., 2021	100 (1.20%)	-	-	22 (22.00%)
Kiran et al., 2021 [42]	504 (6.04%)	-	-	69 (13.69%)
	496 (5.95%)	-	-	11 (2.22%)
Ly et al., 2021 [6]	411 (4.93%)	-	-	37 (9.00%)
Ly et al., 2021 [5]	126 (1.51%)	0 (-)	0 (-)	26 (20.63%)
	111 (1.33%)	0 (-)	0 (-)	0 (-)
	71 (0.85%)	0 (-)	0 (-)	0 (-)
Oette et al., 2021 [100]	130 (1.56%)	-	-	4 (3.08%)
Roland et al., 2021 [103]	1985 (23.80%)	-	-	91 (4.58%)
Oette et al., 2022 [99]	129 (1.55%)	0 (-)	-	4 (3.10%)
	143 (1.71%)	1 (0.70%)	-	0 (-)
	83 (1.00%)	1 (1.20%)	-	5 (6.02%)
Rowan et al., 2022 [102]	871 (10.44%)	-	-	54 (6.20%)
Generaal et al., 2023 [101]	138 (1.65%)	-	-	0 (-)
TOTAL		8/1628 (0.49%)	9/1273 (0.71%)	752/7375 (10.20%)

Note: RSV = respiratory syncytial virus; SARS-CoV-2 = severe acute respiratory syndrome coronavirus 2.

**Table 5:** Occurrence of respiratory viruses in collected specimens from incidence studies.

Study	Sample (N.)	Observation Time (Days)	RSV (n./N, %)	Influenza (n./N, %)	SARS-CoV-2 (n./N, %)
Ralli et al., 2021 [109]	1052	247			86 (8.17%)
Lindner et al., 2021 [111]	118	20			0 (-)
Richard et al., 2021 [112]	721	333			124 (9.66%)
Berner et al., 2022 [113]	11,563	274			903 (7.81%)
Chow et al., 2022 [21]	14,464	608	20 (0.14%)	22 (0.15%)	
Keller et al., 2022 [115]	712	1340			39 (5.48%)
Luong et al., 2022 [116]	4657	105			394 (8.46%)
McCulloch et al., 2023 [20]	825	150	15 (1.82%)	13 (1.58%)	
	15,289	608			133 (0.87%)
Morrone et al., 2023 [110]	5442	579			168 (5.49%)
Rogers et al., 2023 [114]	2360	516			117 (4.96%)
Rogers et al., 2023 [44]	1283	115		51 (3.98%)	
Rogers et al., 2023 [19]	825	346	14 (1.70%)	11 (1.33%)	
TOTAL			49/16,114 (0.30%)	97/17,3997 (0.56%)	1946/41,914 (4.69%)

Note: RSV = respiratory syncytial virus; SARS-CoV-2 = severe acute respiratory syndrome coronavirus 2.

**Table 6:** Detailed reporting of the risk of bias (ROB) estimates for the observational studies [89,119]. Analyses were performed according to the National Toxicology Program’s (NTP) Office of Health Assessment and Translation (OHAT) handbook and respective risk of bias (ROB) tool. Note: D1: possibility of selection bias; D2: exposure assessment; D3: outcome assessment; D4: confounding factors; D5: reporting bias; D6: other bias; ☹☹: definitively high; ☹: probably high; ☺: probably low; ☺☺: definitively low.

Study	D1	D2	D3	D4	D5	D6
Prevalence studies						
Badiaga et al., 2009 [17]	☹	☺	☺	☺	☺	☺☺
Thiberville et al., 2014 [18]	☹	☺	☺	☺	☺	☺☺
Ly et al.,2019 [4]	☹	☺	☺☺	☺☺	☺	☺☺
Baggett et al., 2020 [108]	☹	☺	☺	☹	☺	☺
Imbert et al., 2020 [107]	☹	☺	☺	☹	☺	☺
Karb et al., 2020 [106]	☹	☺	☺	☹	☺	☺
Mosites et al., 2020 [105]	☹	☺	☺	☹	☺	☺
Storgaard et al., 2020 [104]	☹	☺☺	☺	☹	☺	☺
Husain et al., 2021	☹	☺☺	☺	☺☺	☺	☺
Kiran et al., 2021 [42]	☹	☺☺	☺	☹	☺	☺
Ly et al., 2021 [6]	☹	☺	☺	☹	☺	☺☺
Ly et al., 2021 [5]	☹	☺	☺☺	☹	☺	☺☺
Oette et al., 2021 [100]	☹	☺	☺	☹	☺	☺
Roland et al., 2021 [103]	☹	☺☺	☺	☹	☺	☺
Oette et al., 2022 [99]	☹	☺	☺☺	☹	☹	☹
Rowan et al., 2022 [102]	☹	☺☺	☺	☹	☺	☺
Generaal et al., 2023 [101]	☹	☺☺	☺	☺	☺	☺
Incidence studies						
Ralli et al., 2021 [109]	☹☹	☺	☺	☹	☺	☺
Lindner et al., 2021 [111]	☹	☺	☺	☹	☺	☺
Richard et al., 2021 [112]	☹	☺	☺☺	☹	☺	☺
Berner et al., 2022 [113]	☹	☺	☺	☹	☺	☺
Chow et al., 2022a [21]	☹☹	☺	☺	☹☹	☹☹	☹☹
Keller et al., 2022 [115]	☹	☺	☺	☹	☺	☺
Luong et al., 2022 [116]	☹	☺	☺	☹	☺	☺
McCulloch et al., 2023 [20]	☹☹	☺	☺	☹☹	☹☹	☹☹
Morrone et al., 2023 [110]	☹☹	☺	☺	☹	☺	☺
Rogers et al., 2023 [114]	☹☹	☺	☺	☹☹	☹☹	☹☹
Rogers et al., 2023 [44]	☹☹	☺	☺	☹☹	☹☹	☹☹
Rogers et al., 2023 [19]	☹☹	☺	☺	☹☹	☹☹	☹☹
Outcome studies						
Boonyaratanakornkit et al., 2019 [117]	☹☹	☹	☺☺	☺	☺	☺
Loubiere et al., 2023 [118]	☹	☺☺	☺☺	☺	☺	☺

**Table 7:** Summary of pooled prevalence estimates for respiratory viruses included in the analyses. Estimates are reported as a whole and by timeframe, with studies performed before the inception of the SARS-CoV-2 pandemic vs. studies performed since January 2020.

Pathogen	Time Period	Pooled Prevalence (N./1000 Samples, 95%CI)	τ^2;^ (I^2^; 95%CI)
RSV	Overall	4.91 (2.46; 9.80)	0.000 (0.0%; 0.0 to 62.4)
	Pre-Pandemic	6.22 (2.80; 13.77)	0.000 (0.0%)
	Pandemic	3.02 (0.76; 11.98)	0.000 (0.0%)
Influenza	Overall	3.47 (0.47; 25.11)	0.84 (0.0%; 0.0 to 70.8)
	Pre-Pandemic	8.90 (2.82; 27.74)	0.037 (0.0%)
	Pandemic	0.00 (0.00; 1000)	-
SARS-CoV-2	Overall	40.21 (14.66; 105.55)	5.14 (97.5%; 96.9 to 97.9)

**Table 8:** Summary of pooled incidence estimates for respiratory viruses included in the analyses [19,20,21,44,109,110,111,112,113,114,115,116].

Pathogen	Number of Estimates	Number of Events	Pooled Incidence (N./1000 Person-Months, 95%CI)	τ^2;^ (I^2^; 95%CI)
RSV	3	49	1.71 (0.00; 4.13)	0.001 (89.4%; 71.3 to 96.1)
Influenza	4	97	6.07 (0.00; 15.06)	0.001 (95.2%; 90.7 to 97.5)
SARS-CoV-2	9	1964	9.58 (3.00; 16.16)	0.001 (99.1%; 98.9 to 99.3)

**Table 9:** Summary of Egger’s test for publication bias on sampled studies.

Settings	Pathogen	t	df	*p* Value	Bias (SE)	Intercept (SE)
Prevalence	RSV	−1.42	8	0.193	−0.434 (0.305)	−4.592 (0.299)
	Influenza	−8.46	5	< 0.001	−1.159 (0.137)	−3.787 (0.108)
	SARS-CoV-2	−1.28	20	0.216	−2.989 (2.338)	−1.229 (0.476)
Incidence	RSV	21.12	1	0.030	3.708 (0.176)	0.000 (0.000)
	Influenza	3.51	2	0.072	4.940 (1.405)	−0.001 (0.001)
	SARS-CoV-2	2.25	7	0.060	10.994 (4.897)	0.001 (0.001)

## Data Availability

Data are available on request of to the corresponding author.

## Supplementary Materials

The following supporting information can be downloaded at https://www.mdpi.com/article/10.3390/epidemiologia5010004/s1: Table S1: PRISMA Checklist.

## Appendix A

**Table A1:** PECO worksheet [83,84].

Item	Definition
Population of interest	Among individuals being assisted in urban shelters for homeless people,
Exposure	what is the occurrence (i.e., prevalence and/or incidence) of respiratory syncytial virus infection
Control/comparator	in children and adults, compared to influenza virus and SARS-CoV-2
outcome	and the outcome of RSV, influenza and SARS-CoV-2 infections

**Table A2:** Search strategy and number of retrieved entries.

Database	Keywords	N. of Entries
PubMed	(“Respiratory Syncytial Virus, Human” [Mesh] OR “Respiratory Syncytial Viruses” [Mesh] OR “Respiratory Syncytial Virus Infections” [Mesh] OR “RSV” OR “respiratory infection*” OR “respiratory syncytial virus” OR “Influenza, Human” [Mesh] OR “Influenza B virus” [Mesh] OR “Influenza A virus” [Mesh] OR “Severe acute respiratory syndrome-related coronavirus” [Mesh] OR “Middle East Respiratory Syndrome Coronavirus” [Mesh] OR “Nipah Virus” [Mesh] OR “COVID-19” [Mesh] OR “SARS-CoV-2” [Mesh] OR “COVID-19 Testing” [Mesh] OR “Respiratory Tract Infections” [Mesh]) AND (“Ill-Housed Persons” [Mesh] OR “Homeless Youth” [Mesh] OR “homeless”)	797
EMBASE	(“pneumovirus’/exp” OR “pneumovirus” OR “pneumovirus infection” OR “human respiratory syncytial virus” OR “respiratory syncytial virus infection” OR “influenza” OR “influenza virus” OR “influenzavirus a” OR “respiratory virus*”) AND (“homelessness” OR “homeless person” OR “homeless youth”) AND (“prevalence” OR “incidence”)	3210
SCOPUS	(“pneumovirus” OR “pneumovirus infection” OR “human respiratory syncytial virus” OR “respiratory syncytial virus infection” OR “influenza” OR “influenza virus” OR “influenzavirus a” OR “respiratory virus*”) AND (“homelessness” OR “homeless” OR “homeless youth”) AND (prevalence OR incidence)	775
medRxiv	“respiratory virus*” AND “homeless*”	188

**Table A3:** Working definition for homeless people (HP) and homeless shelter.

Item	Definition	Reference
HP	People who do not have access to accommodation which they can reasonably occupy, whether this accommodation is: (i) legally their own property or whether the property is rented; (ii) provided by institutions; (iii) provided by employers; (iv) occupied rent-free under some contractual or other arrangement.	[1]
Homeless Shelter	Temporary residence for HP providing safety conditions and protection from exposure to the weather	[1,4,15,16]
